# Development of Phage Lysin LysA2 for Use in Improved Purity Assays for Live Biotherapeutic Products

**DOI:** 10.3390/v7122965

**Published:** 2015-12-16

**Authors:** Sheila M. Dreher-Lesnick, Jeremy E. Schreier, Scott Stibitz

**Affiliations:** Office of Vaccines Research and Review, Division of Bacterial, Parasitic and Allergenic Products, Center for Biologics Evaluation and Research, U.S. Food and Drug Administration, Silver Spring, MD 20993, USA; Jeremy.Schreier@fda.hhs.gov (J.E.S.); Earle.Stibitz@fda.hhs.gov (S.S.)

**Keywords:** endolysin, live biotherapeutic product, probiotics, purity assay, phage lysins, LysA2, *Lactobacillus*

## Abstract

Live biotherapeutic products (LBPs), commonly referred to as probiotics, are typically preparations of live bacteria, such as *Lactobacillus* and *Bifidobacterium* species that are considered normal human commensals. Popular interest in probiotics has been increasing with general health benefits being attributed to their consumption, but there is also growing interest in evaluating such products for treatment of specific diseases. While over-the-counter probiotics are generally viewed as very safe, at least in healthy individuals, it must be remembered that clinical studies to assess these products may be done in individuals whose defenses are compromised, such as through a disease process, immunosuppressive clinical treatment, or an immature or aging immune system. One of the major safety criteria for LBPs used in clinical studies is microbial purity, *i.e.*, the absence of extraneous, undesirable microorganisms. The main goal of this project is to develop recombinant phage lysins as reagents for improved purity assays for LBPs. Phage lysins are hydrolytic enzymes containing a cell binding domain that provides specificity and a catalytic domain responsible for lysis and killing. Our approach is to use recombinant phage lysins to selectively kill target product bacteria, which when used for purity assays will allow for outgrowth of potential contaminants under non-selective conditions, thus allowing an unbiased assessment of the presence of contaminants. To develop our approach, we used LysA2, a phage lysin with reported activity against a broad range of *Lactobacillus* species. We report the lytic profile of a non-tagged recombinant LysA2 against *Lactobacillus* strains in our collection. We also present a proof-of-concept experiment, showing that addition of partially purified LysA2 to a culture of *Lactobacillus jensenii* (*L. jensenii*) spiked with low numbers of *Escherichia coli* (*E. coli*) or *Staphylococcus aureus* (*S. aureus* ) effectively eliminates or knocks down *L. jensenii,* allowing for clear detection of the contaminating strains. With continued identification and characterization of phage lysins, we hope that the use of recombinant phage lysins in purity assays for products containing live microbials may offer additional tools to help advance product development of LBPs.

## 1. Introduction

Our knowledge of, and interest in, the human microbiota is currently undergoing an explosion fueled by the convergence of technological advances, such as high-throughput DNA sequencing, and research developments that have demonstrated the importance of our microbial flora in both health and disease. In the public domain, this interest has been echoed by the availability of a large variety of over-the-counter (OTC) probiotics. However, a quick perusal of ClinicalTrials.gov [1] indicates several studies that test specific probiotic products for their effectiveness in treating specific diseases, often in individuals whose health is compromised for various reasons. As such, probiotics meet the definition of a drug (any product that is intended for use in the diagnosis, cure, mitigation, treatment, or prevention of disease) according to section 201(g)(1)(B) [21 U.S.C. 321 (g)(1)(B)] of the Food, Drug, and Cosmetic Act [2], placing them under the purview of the Center for Biologics Evaluation and Research (CBER), and human clinical trials must be pursued through the Investigative New Drug (IND) process. In 2012, CBER published a guidance document [3], which defines products commonly referred to as probiotics as live biotherapeutic products (LBPs).

Federal regulations governing the IND process mandate sufficient CMC (Chemistry, Manufacturing, and Control) documentation to assure that products administered to human subjects are safe [3,4]. Since the overriding concern in early clinical trials with LBPs is safety, adequate microbiological testing of such products is crucial. Microbial purity, *i.e.*, the presence of no more than a small number of extraneous microorganisms, and the absence of pathogenic microorganisms, that may be introduced during various stages of manufacture and product handling, is a major issue in LBP testing. Devising adequate and feasible methods for demonstrating microbial purity is a challenge, because the presence of large numbers of viable bacteria in such products can overwhelm standard culture techniques. To address this challenge, we have been working to develop new reagents that can, with high specificity, selectively kill, or inhibit the growth of product organisms, while allowing for the outgrowth of a variety of potential contaminants in an unbiased fashion. The availability of such reagents will allow the comprehensive microbiological analysis using standard techniques without interference. In our lab, we have been working to develop recombinant phage lysins as reagents for use in purity and potency assays for LBPs.

Phage lysins of bacteriophages that infect Gram-positive bacteria are peptidoglycan hydrolases, that typically consist of one or two N-terminal enzymatic domains and a C-terminal cell wall binding domain (CBD). Lysins are produced towards the end of the phage replication cycle in conjunction with holin proteins. The holin creates a pore in the inner membrane, allowing the lysin access to the peptidoglycan, where it can exert its enzymatic function and ultimately cause lysis of the cell. “Lysis from without” can occur when lysin is introduced to peptidoglycan externally [5]. Lysin CBDs encoded by bacteriophage of Gram-positive bacteria confer specificity to the enzyme, allowing attachment to the peptidoglycan of target bacteria, with some showing lysin activity confined to the host genus or species [6,7]. Once in close proximity, the enzymatic domain is able to exert its function to break down the peptidoglycan, and lead to rapid lysis of the cell [8]. According to Fischetti, 2008 [6], a single lysin molecule should be catalytically effective in lysing Gram-positive peptidoglycan. Presumably due to the ability of the lysin to bind to and cleave essential and conserved structures in peptidoglycan, resistance appears to be a very rare event, making lysins attractive candidates for use as antimicrobials and tools for microbial detection [6].

In this study, we used LysA2, an endopeptidase originating from the *Lactobacillus casei* (*L. casei*) bacteriophage A2, which is able to hydrolyze the bond between the terminal D-alanine of the peptidoglycan tetrapeptide and the aspartic acid residue that creates the bridge with the L-lysine of the adjacent peptidoglycan chain [7]. This lysin has been found to act on a wide range of lactic acid bacteria (LAB) characterized by Gram-positive bacteria in the A4 peptidoglycan subgroup. Additionally, LysA2 is catalytically active at a wide range of temperatures, does not require a reducing medium, is able to maintain activity in a broad range of pH values with optimal activity at pH 5–5.5, and has activity independent of divalent cations [7]. All these characteristics make LysA2 a viable candidate in our assay development.

LysA2 was synthesized and cloned into a low copy, arabinose-inducible expression vector (pNW129). Untagged, full-length recombinant protein was expressed in *E. coli* BL21 cells, and subsequently tested for lytic activity in a 96-well turbidometeric assay (adapted from Schuch *et al.*, 2009 [9]). We examined the lytic activity of the A2 recombinant protein against strains in our collection, and chose to use *L. jensenii* as our target strain for further development of this assay. Full length LysA2 was added to test cultures that had been spiked with organisms representing potential contaminating bacterial pathogens. Our intent is to kill the target (product) strain, allowing for the detection of contaminating organisms.

## 2. Materials and Methods

### 2.1. Bacterial Strains and Cloning

The nucleotide sequence of the A2 lysin (LysA2) was obtained from GenBank (Nucleotide accession #AJ251789; Protein accession #NP_680500; NCBI; see supplementary materials). The sequence was codon optimized for *E. coli*, and synthesized and cloned into the pNW129 low copy expression plasmid (received from Deborah M. Hinton, NIDDK, NIH) by Genscript USA to make pSDL129. For recombinant protein expression, pSDL129 was transformed into *E. coli* BL21 DE3. Clone candidates were verified by both sequencing the insert (Macrogen, Rockville, MD, USA) and by digesting with EcoRI and XbaI (NEB). Several Lactobacillus species from our collection, including some species commonly found in OTC probiotic products, were used in our lytic activity screen. All strains used in our experiments are summarized in Table 1 below:

**Table 1:** List of strains used in our lytic activity screen and mock purity/spiking assay.

Strain	Source
*Lactobacillus gasseri* 33323	ATCC
*Lactobacillus casei* 393	ATCC
*Lactobacillus jensenii* 25258	ATCC
*Lactobacillus plantarum* (V)	product strain
*Lactobacillus delbrueckii* sub lactis 15808	ATCC
*Lactobacillus gasseri* P1	product strain
*Lactobacillus gasseri* P2	product strain
*Lactobacillus gasseri* P3	product strain
*Lactobacillus* *gasseri* P4	product strain
*Lactobacillus reuteri*	product strain
*Lactobacillus acidophilus* (V)	product strain
*Lactobacillus paracasei* (V)	product strain
*Lactobacillus plantarum* (D)	product strain
*Lactobacillus rhamnosus* (D)	product strain
*Lactobacillus vaginalis* 49540	ATCC
*Lactobacillus lactis* 1154	ATCC
*Lactobacillus johnsonii* 11506	ATCC
*Escherichia coli* 86-24 (O157 H7) (plasmid cured)	kindly provided by Allison O’Brien
*Staphylococcus aureus* N315	kindly provided by Mark Shirtliff

### 2.2. Protein Expression and Purification

A 5 mL culture of *E. coli* BL21 DE3 containing the pSDL129 plasmid was grown under shaking overnight at 37 °C in Luria Bertani broth (LB; BD Difco, Sparks, MA, USA) plus Kanamycin (40 µg/mL). The sample was sub-cultured the following morning at 1:100 into 50 mL LB Kan. After 2 h of growth at 37 °C (OD_600_~0.3), arabinose was added to a final concentration of 0.2% and the culture was left to shake for another 2 h. Cells were harvested and spun at 2600× *g* for 20 min at 4 °C (Sorvall Legend RT, Kendro Laboratory Products, Germany). The supernatant was discarded and the pellet was stored at −80 °C. The cell pellet was resuspended in 4 mL 25 mM MES pH 5.5 and cells were broken by sonication using six 15 s pulses with intervening 10 s rest periods, on ice (Misonix microson ultrasonic cell disruptor XL, Misonix Incorporated, Farmingdale, NY, USA). Cell debris was removed by centrifugation (2600× *g*, 25 min, 4 °C) and the supernatant sterilized by passage through a 0.45 µm filter. Volume was reduced and smaller solutes removed using an Amicon Ultra-4 centrifugal filter Ultracel-30K (Millipore, Billerica, MA, USA). The filtrate was discarded and the retentate was passed through an Amicon Ultra-4 centrifugal filter Ultracel-50K (Millipore) for partial purification. The filtrate was placed on ice until needed.

Further purification was achieved by following the same protocol to the point following the 0.45 µm filter at which point the sample was applied to Pierce Strong Anion Exchange Spin Columns, Maxi (Thermo Scientific, Waltham, MA, USA). Columns were washed with 25 mM MES pH 6.0 and centrifuged (200× *g*, 5 min, 4 °C). Supernatant was then added to the column and centrifuged (200× *g*, 5 min, 4 °C). The eluate, containing the recombinant lysin protein was collected and buffer exchanged into 25 mM MES pH 5.5 using Amicon Ultra-4 Centrifugal Filters Ultracel-10K (Millipore). To determine the lytic activity of fractions and the crude lysate, they were diluted 10-fold and 100-fold and tested on *L. jensenii* according to the lytic assay below. Samples were checked for purity on a NuPAGE 4%–12% Bis-Tris Gel 1.5 mm × 15 well (Novex, by Life Technologies, Carlsbad, CA, USA)) with a SeeBlue Plus 2 prestained ladder (Novex), and visualized using Instant Blue (Expedeon Inc., San Diego, CA, USA). Protein concentration of LysA2 was determined using a Pierce BCA Protein Assay Kit (Thermo Scientific).

### 2.3. Lytic Assay

*Lactobacillus* cells were grown overnight at 37 °C in 5 mL de Man, Rogosa, Sharpe (MRS) broth (Oxoid CM0359) under anaerobic conditions (Whitley workstation DG250; Microbiology International, Frederick, MD, USA). Cells were subsequently sub-cultured and grown to mid-exponential phase (OD_600_ range of 0.3–0.8), pelleted, and washed twice in an equal volume of 25 mM MES pH 5.25. The OD_600_ was assessed prior to the second wash step and adjusted to a final OD_600_ of 1.0 following re-suspension. Activity of the purified fractions was compared to crude lysate containing LysA2. Crude lysates correspond to the preparations prior to anion exchange purification. Total protein of the lysate was determined using a Pierce BCA Protein Assay Kit (Thermo Scientific).

The turbodimetric lytic assay was adapted from Schuch *et al.*, 2009 [9]. *Lactobacillus* cells (160 µL) were incubated with 40 µL of LysA2 lysate or control (25 mM MES pH 5.25), and immediately placed into a Cytation 3 Imaging Reader (Biotek Instruments Inc., Winooski, VT, USA) set at 37 °C with continuous shaking. OD_600_ was measured every minute for up to 2 h using the Gen5 v2.06 program (Biotek). Data were exported to Excel, and specific activity was calculated by determining the greatest slope in the linear portion of the curve with the highest R^2^ value for the change in OD_600_ min^−1^, and values were normalized to total protein content of the pellet. Following the completion of the assay, 10 µL of sample from each well was spotted onto MRS agar plates and allowed to dry before plates were incubated at 37 °C for up to two days. All assays were carried out in triplicate.

### 2.4. Mock Purity and Spiking Assay

**Figure 1:**
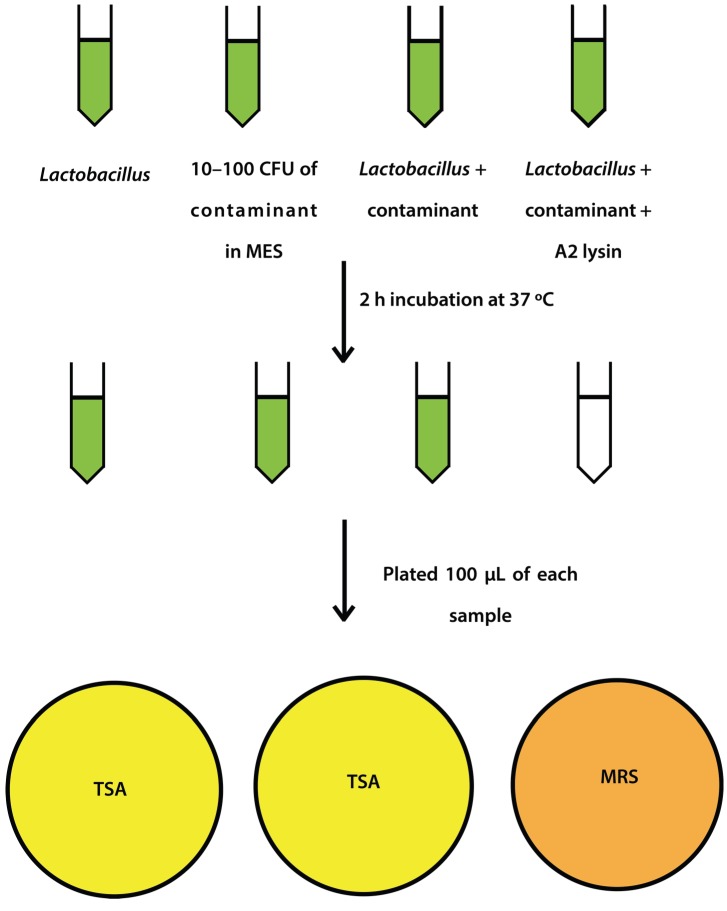
Schematic representation of spiking experiment.

*L. jensenii* cells were grown in 5 mL MRS overnight at 37 °C anaerobically, while *E. coli* 86-24 and *S. aureus* N315 were grown in 5 mL TSB overnight aerobically at 37 °C. All samples were sub-cultured in their respective broth the following morning to an OD_600_ of ~0.2. *L. jensenii* was grown in 3 separate tubes of 5 mL MRS anaerobically at 37 °C to mid-exponential phase (OD_600_ 0.4–0.6; 1 × 10^4^–1 × 10^5^ CFU/mL) while *E. coli* and *S. aureus* were grown at 37 °C to an OD_600_ of 0.6–1.0. *L. jensenii* cells were then washed twice in 25 mM MES pH5.5 (spun 2600× *g*, 10 min, 4 °C) and 2 tubes were re-suspended in 6 mL 25 mM MES pH 5.5 and 1 tube was re-suspended in 5 mL 25 mM MES pH 5.5. *E. coli* or *S. aureus* cells (10–100 CFU) were added to 6 mL 25 mM MES pH 5.5, and a 5 and 6 mL tube of *L. jensenii* in 25 mM MES pH 5.5. One mL of partially purified LysA2 was added to the 5 mL tube of *L. jensenii* with *E. coli* or *S. aureus* to a final volume of 6 mL. All tubes were immediately placed at 37 °C with shaking for 2 h with OD_600_ readings taken prior to incubation. After incubation, OD_600_ readings were taken again and 100 µL of each tube was spread onto 2 TSA plates and 1 MRS plate (Figure 1). All plates were placed at 37 °C in an anaerobic chamber until growth was observed on all plates (up to 2 days for *L. jensenii* growth). Plates were imaged using a Color Q Count (Spiral Biotech, Norwood, MA, USA).

## 3. Results

### 3.1. Lytic Activity

**Figure 2:**
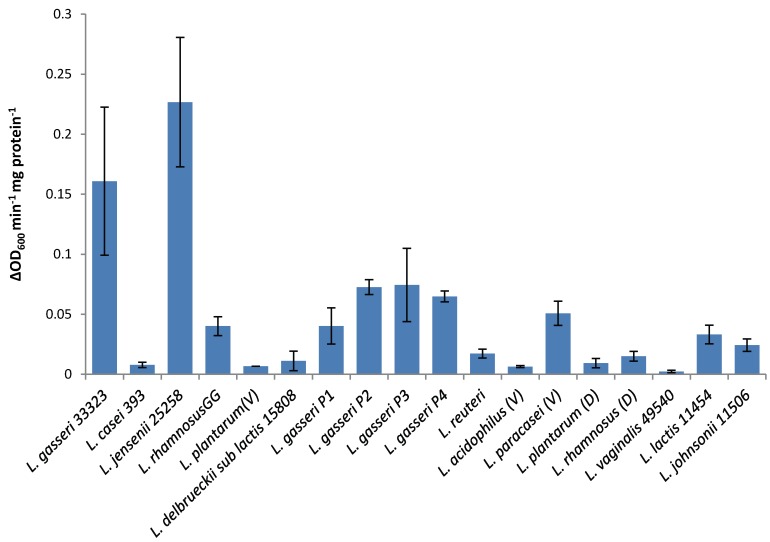
Specific activities of LysA2 against different *Lactobacillus* sp. Specific activity was calculated as described in Material and Methods. Strains are listed in Table 1. All experiments were performed in triplicate and error bars indicate standard error.

As reported elsewhere [7], recombinant LysA2 has a broad range of activity on the *Lactobacillus* species tested in our collection (Figure 2). *L. jensenii* and *L. gasseri* (ATCC strain) had the fastest observable lytic activity for LysA2. Lysis was also observed in other species such as *L. johnsonii, L. lactis L. rhamnosus GG*, *L. gasseri* (product strains) and *L. paracasei* however it was more subtle, either characterized by a slow, linear decrease in OD_600_ over the span of the 2 h period, or a quick, short decrease in OD_600_ that was unable to lyse the culture to a considerable extent. Other species, such as *L. casei, L. vaginalis, L. reuteri, L. rhamnosus* (D), *L. plantarum* (V), *L. plantarum* (D), *L. acidophilus* (V), *L. delbrueckii sub lactis*, and *L. acidophilus* had little to no discernable susceptibility to LysA2’s lytic activity. A more objective determination of the lytic activity was obtained by calculating specific activity as the rate of decrease in absorbance (change in OD_600_ min^−1^) per unit of lysin preparation (mg), with the rate determined from the slope of the most linear portion of the curve.

It was found that the specific activity observed against different strains did indeed correspond to the more subjective descriptions given above. *L. jensenii* and *L. gasseri* have the highest lytic activity at 0.227 and 0.161 ΔOD min^−1^ mg·protein^−1^, respectively, and these strains displayed the most dramatic drops in OD_600_ (Figure 2). *Lactobacillus* species such as *L. johnsonii, L. lactis L. rhamnosus GG, L. gasseri* (product strains) and *L. paracasei* that displayed moderate lysis by LysA2 had specific activities from 0.0243 to 0.0743 ΔOD min^−1^ mg·protein^−1^. Species that displayed little to no lysis, such as *L. casei, L. vaginalis, L. reuteri, L. rhamnosus (D), L. plantarum* (V), *L. plantarum* (D), *L. acidophilus* (V), *L. delbrueckii sub lactis,* and *L. acidophilus* showed specific activities from 0.00226 to 0.0171 ΔOD min^−1^ mg·protein^−1^. An unexpected result was that the recombinant LysA2 did not demonstrate activity against its reported host strain, *L. casei* ATCC 393 (Figure 2).

**Figure 3:**
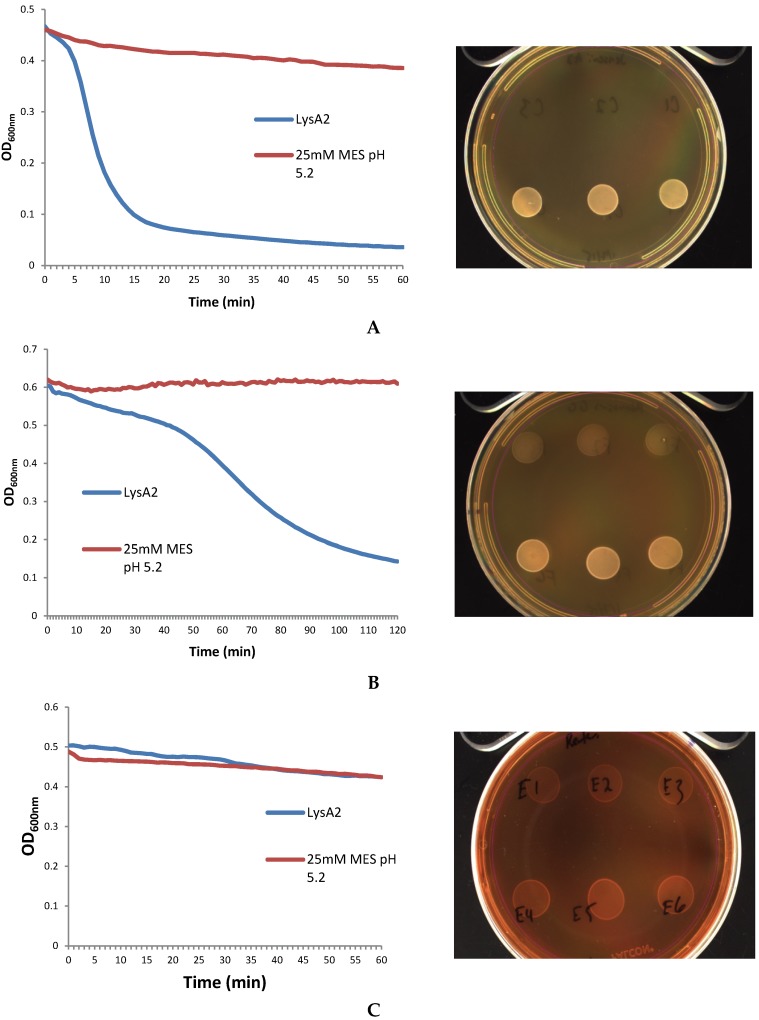
Correlation of lytic activity of LysA2 determined by reduction in OD_600_ to cell viability on MRS plates. Samples from triplicate wells (experimental on the top row and buffer only controls on the bottom row) used in the lytic assay were spotted onto MRS agar (one spot from each well) and incubated for up to two days to determine cell viability. Images of *Lactobacillus* spots are shown to the right of the corresponding lytic assay. LysA2 lytic activity can be divided into three categories: (**A**) strong lytic activity of LysA2 on *L. jensenii*; (**B**) oderate lytic activity of LysA2 on *L. rhamnosus GG*; and (**C**) no lysis of *L. reuteri* by LysA2.

In our cell viability spot assays, where 10 µL of the co-incubated samples from the lytic assay were subsequently spotted onto MRS agar and incubated for two days at 37 °C, a significant decrease in lawn density was observed compared to the buffer only control (Figure 3A) Spots from the *L. jensenii/*LysA2 mixture are clear with only few persisting colonies visible, compared to buffer control. On the other hand, spots of the *L. rhamnosusGG/*LysA2 mixture showed a less dramatic reduction in cell clearing compared to *L. jensenii*, but did still show a reduction in the density of the lawn (Figure 3B). The mixture of *L. reuteri*/LysA2 showed no decrease in OD_600_, coinciding with no difference between lysin and buffer treated spots (Figure 3C). Based on the combined results of our turbidometric assay and viability assay, we chose to use *L. jensenii* as the target strain for further development of this assay.

### 3.2. Protein Purification

**Figure 4:**
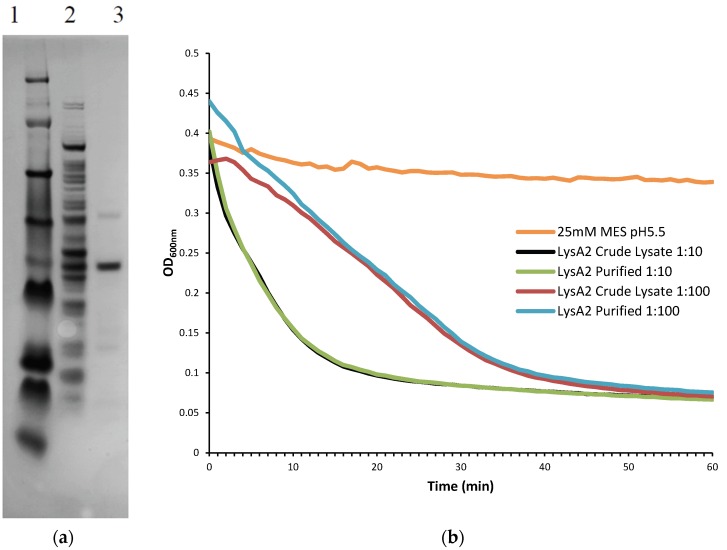
(**A**) Purification of LysA2 (37.4 kDa) by anion exchange. SDS-PAGE gel for purified LysA2 samples that had been diluted 10-fold Lanes 1—See Blue Plus2 Protein Ladder; Lanes 2—LysA2 crude lysate; Lanes 3—Purified LysA2; (**B**) Ten- and 100-fold diluted purified LysA2 and crude lysate in a turbidometric reduction assay against *L. jensenii*. Activity of the purified and crude LysA2 display the same kinetics for both dilutions tested.

Using an anion exchange column equilibrated with 25 mM MES pH6.0, the purified LysA2 was collected from the flow-through fraction (Figure 4A). When activity of this fraction was tested on *L. jensenii*, we found that the purified LysA2 and crude lysate displayed the same lytic kinetics and activity when diluted by 10- and 100-fold (Figure 4B). While observed lytic activity between the two samples was similar, normalizing to their protein content indicated an increase in specific activity of the purified fraction relative to the crude lysate. Calculating using the data from the 100-fold dilutions, specific activity of the crude lysate was 10.5 ΔOD_600_ min^−1^ mg·protein^−1^, while that of the purified LysA2 fraction 74.1 ΔOD_600_ min^−1^ mg·protein^−1^.

### 3.3. Mock Purity Assay and Spiking Experiments

**Figure 5:**
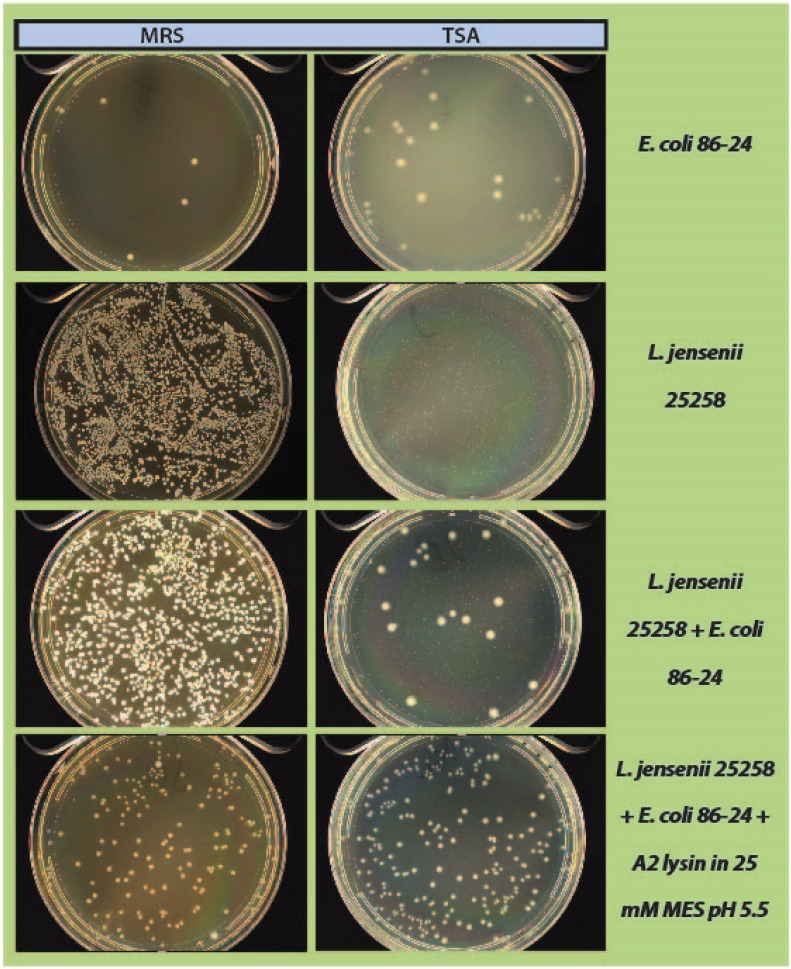
Mock purity assay, using *L. jensenii* as a mock product strain, and spiked with *E. coli* as a contaminant. High density *L. jensenii* cells were spiked with low numbers of *E. coli*. Samples incubated with LysA2 showed no *L. jensenii* growth, allowing for the clear recovery and detection of contaminating *E. coli* colonies.

A proof-of-concept experiment was carried out by spiking in low numbers of a known Gram-positive or Gram-negative contaminant to a high density culture of *L. jensenii,* using LysA2 to eliminate or knock-down *L. jensenii*, and thus enable efficient recovery of the contaminant. Two types of media were used for plating to examine the effect of LysA2 activity on growth of *L. jensenii* and the contaminant strains when exposed to optimal and sub-optimal conditions. When *L. jensenii* cultures (10^4^–10^5^) were spiked with a low number of *E. coli* (10–100 CFU), incubated with LysA2 for 2 h, and subsequently plated on MRS and TSA plates, we observed a marked decrease of *L. jensenii*, with no effect on the growth of *E. coli* (Figure 5). Treatment with LysA2 allowed for recovery and clear distinction of *E. coli* colonies from *L. jensenii* colonies on both MRS agar and TSA agar. Our results for the assay using MRS agar, which is an optimal growth medium for *L. jensenii,* clearly show how addition of LysA2 to the mixture effectively eliminates the mock product strain, while allowing for detection of low numbers of contaminating *E. coli*. Without addition of LysA2, *E. coli* colonies cannot be distinguished among the high density growth of *L. jensenii*. When mixtures were plated on TSA agar, a suboptimal growth medium for *L. jensenii*, *E. coli* colonies could more clearly be distinguished from *L. jensenii*, with the latter forming much smaller colonies compared to *E. coli*. To confirm the identities of the larger colonies on these plates, representative ones were picked and grown on MRS and TSA under aerobic and anaerobic conditions. Colonies grew on both types of media in both conditions, indicating that they were *E. coli*, since *L. jensenii* does not grow under aerobic conditions. When mixed samples treated with LysA2 and subsequently plated on TSA, only *E. coli* colonies were detected, with no visible growth of *L. jensenii.*

**Figure 6:**
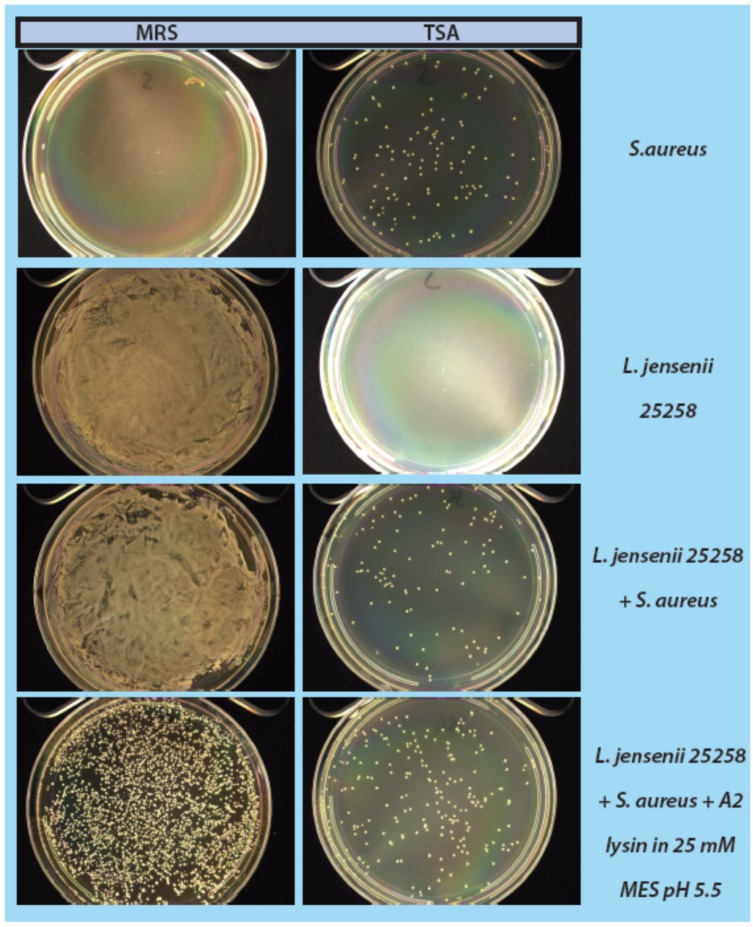
Mock purity assay, using *L. jensenii* as our mock product strain, and spiked with *S. aureus* N315 as a contaminant. High desnity *L. jensenii* cells were spiked with low numbers of *S. aureus* N315. Samples incubated with LysA2 showed *L. jensenii* growth, allowing for the clear recovery and detection of contaminating *S. aureus* colonies. Samples treated with LysA2 and plated on TSA showed fewer background colonies of *L. jensenii* (barely visible pinpoint colonies) compared to samples not treated with LysA2. The larger colonies seen on the TSA plates post LysA2 treatment are all *S. aureus*. The identical sample plated on MRS agar does not allow for easy distinction of *S. aureus* from *L. jensenii*.

When *S. aureus* was used as a contaminant to spike in the mock purity assay, the observed results were not as clean as observed for the assay when spiking with *E. coli* (Figure 6). *S. aureus* had some difficulty growing on MRS medium, but showed some growth (~30–50 colonies) on TSA. The addition of LysA2 to the *L. jensenii + S. aureus* mix knocked down growth of *L. jensenii* from a confluent lawn to individual colonies clearly distinguishable from *S. aureus.* This pattern was consistently seen on both TSA and MRS growth media (Figure 6).

## 4. Discussion

As OTC probiotic supplements enter clinical trials to investigate their potential use as therapeutics for various disease conditions (see clinicaltrials.gov for a listing), more stringent quality controls need to be in place to ensure purity and potency of the product [3]. While these products, sold primarily as dietary supplements in the U.S., are considered safe in healthy populations, additional considerations and controls are necessary in products planned for use in vulnerable populations. Product purity is defined as the absence of extraneous, potentially harmful orgnanisms. A recent fatal case of mucormycosis linked to a contaminated OTC probiotic supplement administered to a preterm infant [10], highlights the importance of determining product purity of LBPs when used in such vulnerable patient populations. Current culture-based assays to assess purity, such as those outlined in USP Pharmacopeia <61> and <62> [11,12] can be confounded by the presence of a high number of product organisms. Our goal is to develop a simple yet robust assay using phage lysins to eliminate or knock-down product organisms and allow the outgrowth and detection of potentially harmful contaminants.

Interest in recombinant bacteriophage lysins has greatly expanded in recent years with prospective uses ranging from alternatives to standard antibiotics, to use in agriculture, food safety, industry, and biotechnology [5]. This interest was greatly enhanced with the apparent ability to use lysins “from without”, their specificity, and reports that resistance does not emerge [5,6,8,13]. Some recombinant phage lysins have also been found to have high thermostability [14], as well as ease of expression and genetic manipulation for domain swapping and tagging to alter binding affinity or range of activity [15,16,17]. Initial work showing the application of lysins as antimicrobials was pioneered by Schuch *et al.*, 2002 [18], who identified recombinant phage lysins that effectively lyse *Bacillus anthracis* when applied exogenously in a mouse model. Since then, subsequent studies from the same group and others [19,20,21] have shown the potential for the use of recombinant lysins as alternatives to antibiotics. One example is that of McCullers *et al.*, 2007 [22], who used purified Cpl-1 lysin to effectively prevent acute otitis media in mice, inhibiting secondary bacterial infection following influenza infection, with no apparent toxicity of illness. Similar results for use of lysins as antimicrobials are reported in studies for other infections with potential applications against *Staphylococci* [23,24] and *Streptococci* [19,20].

Other studies propose using whole recombinant endolysins as biopreservatives in food products [25]. Purified phage endolysin LysH5 from a *Staphylococcus aureus* phage was able to inhibit growth of low levels (10^3^ CFU/mL) of *S. aureus* when added to pasteurized milk [25]. Additionally, a synergistic effect was observed when LysH5 was used with nisin, a bacteriocin already in use as a biopreservative in food. Nisin alone inhibited cellular growth, but when used with LysH5, a complete elimination of *S. aureus* was observed in the milk after 6h of incubation [26]. Applications of recombinant lysins as antimicrobials have been primarily against Gram-positive organisms, which due to their cell wall structure, allow for exogenous application of the lysins. More recently, however, some applications of recombinant lysins to target Gram-negative bacteria have also been investigated. Current advances in applying endolysins against Gram-negative bacteria are extensively reviewed by Briers and Lavigne, 2015 [27].

Some other proposed applications for lysins include use of recombinant and chimeric lysins in biopreservation of foods or in food safety applications [24]. Several studies have published successful use of chimeric proteins, where the CBD is used for specificity, and is tagged or labeled for use as tools for detection. Work on *Listeria* endolysins has shown that high affinity CBDs are able to efficiently discriminate and tag different strains of *Listeria monocytogenes* using fluorescently tagged *Listeria* phage endolysins [16].

Use of lysins has also been proposed in other industry applications, such as during the fermentation process of biofuels [28]. LAB are generally considered the most harmful contaminants in fuel ethanol production requiring treatment with antibiotics or expensive disinfecting procedures to alleviate the problem. Roach *et al.*, 2013 [28] provides evidence that LysA2, and other lysins, can be used as antimicrobials to effectively reduce or eliminate LAB, specifically *Lactobacillus* sp. contamination. Our approach is similar to that proposed by Roach *et al.*, 2013 [28], where our goal is to use LysA2 in a purity assay for LBPs to target and remove or knock-down product lactobacilli species to allow the outgrowth of potential contaminating pathogenic bacteria.

The lytic screen of our LysA2 protein showed similar patterns to previously published results [7,28]. Differences in observed activity against different species of *Lactobacillus* could be attributable to the fact that different strains of the same species were used in the different studies. In our study, we observed different activity kinetics for two different strains of *L. rhamnosus* (see Figure 2). Other possible explanations for differences in activity could include differences in recombinant protein constructs, such as the presence or absence of affinity tags, and codon optimization of the sequence for *E. coli*. While all published lytic data for LysA2 is from studies using turbidometric based lytic assays, differences in protocols could also affect observed differences in lytic activity. Unlike Ribelles *et al.*, 2012 [7], we performed lytic assays on whole cells. In addition, unlike Roach *et al.*, 2013 [28], we used MES at pH 5.25–5.5. These observed differences highlight the importance of a thorough characterization of lytic activity for each recombinant lysin in conditions appropriate for its application.

As a step in developing improved microbial purity assays for LBPs, we have demonstrated that LysA2 can effectively eliminate large numbers of *L. jensenii* cells spiked with 10–100 CFU of either *E. coli* or *S. aureus*. Addition of LysA2 to the mixture allowed for clear identification of *E. coli* and *S. aureus* colonies during subsequent plating on MRS and TSA agar. Without addition of the lysin, *E coli* and *S. aureus* colonies are difficult to distinguish on MRS agar, highlighting the potential problem of product interference in detecting small numbers of contaminating organisms. Incubating with lysin, in addition to plating on agar that is more conducive to growth of non-lactobacilli, can be an effective method for detecting low numbers of contaminating bacteria in the presence of a dense population of product bacteria. Additional optimization is necessary to develop LysA2 and other lysins as robust reagents for use in purity assays for LBPs. This will include determination of appropriate storage conditions, stability testing, and range of application for various species in a variety of buffers and media. Activity will need to be fully characterized for both the target organism as well as potential contaminants that we are hoping to detect. Ultimately, purity assays may involve the use of multiple lysins that may act synergistically for optimum elimination of large number of product bacteria, without affecting the growth of any potential contaminants. We feel that development of lysins as tools and reagents in purity assays for LBPs show great promise. We anticipate that using recombinant lysins in culture based assays to evaluate purity of LBPs will allow to the development of simple yet robust assays to detect both Gram-positive and Gram-negative contaminating bacteria, without the use of multiple different selective media.

## 5. Conclusions

This study presents data for our approach to improve purity testing of live biotherapeutic products (LBPs) using recombinant phage lysin LysA2. Our intent is to use recombinant LysA2 to selectively kill the product organism and allow for outgrowth and detection of potential contaminants. Full-length recombinant LysA2 was expressed in BL21cells, tested for lytic activity against our *Lactobacillus* strain collection, and subsequently used in a mock purity assay. In our experiments for the mock purity assay, we used *L. jensenii* to represent our product strain, and we used *E. coli* and *S. aureus* as representative contaminants. We have demonstrated that addition of recombinant LysA2 can effectively kill *L. jensenii* and allow for the detection of low numbers (10–100 CFU) of spiked-in *E. coli* or *S. aureus*. While additional optimization is necessary, the development of phage lysins as reagents for use in purity assays for LBPs shows great promise.

## Supplementary Files

Supplementary File 1
