# Supplementary material for: Development of Phage Lysin LysA2 for Use in Improved Purity Assays for Live Biotherapeutic Products
Source: Viruses. 2015 Dec 16;7(12):6675–88. doi: 10.3390/v7122965 (PMC4690888; doi:10.3390/v7122965)
Supplement: Supplementary File 1 [file viruses-07-02965-s001.pdf]

Supplementary Materials: Development of Phage Lysin LysA2 for Use in Improved Purity Assays for Live Biotherapeutic Products

Sheila M. Dreher-Lesnick, Jeremy E. Schreier and Scott Stibitz

Accession #AJ251789

GAATTCCTTTAAGAAGGAGATATACCATGTCCTACACCATCAATAAAGAATTTGCCCTGGGCGCT
AATGAAGGCTCGTCACAAGTTGCTAACCGTCTGTATATCATCCTGCATGATGTTGGTGCAGAAAG
CGGTGCACGTGCTAACGCAGCATACTTCAAAAACAACATCTCTGCGGAAATCGCCTACACGGCA
TTCGTGGTTGGTGATGGCGGTCAGGTTTATCAAGTCGGTGAACCGGGTTACGTGCAGTGGGGTGC
AGGTACCGTTGCAAACGCCAATTCACCGGTCCAAATTGAACTGGGTCATACGTCGGATCCGGAA
ACCTTTAAAAAAGACTATGCGGTCTACATTGAACTGGCCCGTGATATGGCAGCTCGCTATGGCAT
CCCGACGTCACTGGACGCTGGCGGTGCAGGTACCCCGGGTATTAAATCGCACCTGTGGGTGACG
CAGCATATCTGGGGTGATCACACCGACCCGTATGGTTACCTGGCGCGTTGGGGCATCACGAAAG
AAAAACTGGCAGCAGATCTGGCAAACGGTACCACGACCGTTGACGCCAGCACCTCTGCACCGGC
TACGCAGAGTACCCGTCCGCAAGCAACGGTCTCCGGTAACGTGAATGCTACCTATGGCCTGCATC
TGCTGGGCGGTAGCTGGCTGGATGAAGTGACCAACTTTGGCTCTGGTGACAATGGCTTCGCGGGT
ATGCCGAATCATCAGCACGATCTGCTGTATATTCGTGTTGACCATGGTAGTGTCAAATACCGCGT
GCACACCGTTCAATCCGGTTGGCTGGATTGGGTGACGAAAGGCGATCGCAACGACACCGTTAAT
GGCTGCGCCGGTATTGCAGGCGAAGCTATCGATGGTGTGCAGATTATCTTCCTGACCCCGGCAGG
CGAACCGTATCAGCAAGCTTATTACCGTTCACAGACGACCCAACGTGCAGGTTGGCTGGGTGTCG
TGTGTGATGACGGCACCTCCCTGCCGCAGTATACGGGTACCTACGCAGGCCTGTTTGGTGAACCG
CTGGACCGTCTGCAAATCGGCATCTCGTCAATCAACCCGTTTTAAATCTAGA

Accession #NP_680500

MSYTINKEFALGANEGSSQVANRLYIILHDVGAESGARANAAYFKNNISAEIAYTAFVVGDGGQVYQV
GEPGYVQWGAGTVANANSPVQIELGHTSDPETFKKDYAVYIELARDMAARYGIPTSLDAGGAGTPGIK
SHLWVTQHIWGDHTDPYGYLARWGITKEKLAADLANGTTTVDASTSAPATQSTRPQATVSGNVNAT
YGLHLLGGSWLDEVTNFGSGDNGFAGMPNHQHDLLYIRVDHGSVKYRVHTVQSGWLDWVTKGDRN
DTVNGCAGIAGEAIDGVQIIFLTPAGEPYQQAYYRSQTTQRAGWLGVVCDDGTSLPQYTGTYAGLFGE
PLDRLQIGISSINPF

© 2015 by the authors; licensee MDPI, Basel, Switzerland. This article is an open access article distributed under the terms and conditions of the Creative Commons by Attribution (CC-BY) license (http://creativecommons.org/licenses/by/4.0/).
